# Severe aortic regurgitation with acute decompensation as initial presentation of Behçet’s syndrome: a case report

**DOI:** 10.1093/ehjcr/ytaf567

**Published:** 2025-10-31

**Authors:** Sohaïb Mansour, Ivan Dimov, Michaël Rietz, Abraham J Beun, Philippe Unger

**Affiliations:** Department of Cardiology, CHU Saint-Pierre, Université Libre de Bruxelles, Rue aux Laines 105, Brussels 1000, Belgium; Department of Cardiology, CHU Saint-Pierre, Université Libre de Bruxelles, Rue aux Laines 105, Brussels 1000, Belgium; Department of Cardiology, CHU Saint-Pierre, Université Libre de Bruxelles, Rue aux Laines 105, Brussels 1000, Belgium; Department of Internal Medicine, CHU Saint-Pierre, Université Libre de Bruxelles, Rue aux Laines 105, Brussels 1000, Belgium; Department of Cardiology, CHU Saint-Pierre, Université Libre de Bruxelles, Rue aux Laines 105, Brussels 1000, Belgium

**Keywords:** Behçet’s syndrome, Aortic regurgitation, Acute aortic pseudoaneurysm, Case report, Bentall surgery, Echocardiography, Immunosuppressive therapy

## Abstract

**Background:**

Behçet’s syndrome (BS) is a rare, chronic multisystem inflammatory disorder that can lead to severe cardiovascular complications. Among these, aortic pseudoaneurysms are infrequent and associated with high mortality due to their risk of rupture. The diagnosis of BS remains challenging due to its variable clinical presentation and the absence of specific biomarkers, particularly when the initial presentation is atypical.

**Case summary:**

We present the case of a 32-year-old former professional football player who presented with severe aortic regurgitation with heart failure due to a pseudoaneurysm of the ascending aorta. Initial suspicion of infective endocarditis and aortic dissection was excluded after a clinical, microbiological, and imaging assessment. Emergent Bentall procedure was successfully performed. Subsequently, typical clinical features, including recurrent oral ulcers, pseudofolliculitis, and superficial venous thrombophlebitis, led to the diagnosis of BS. The patient received systemic immunosuppressive therapy combining corticosteroids and infliximab. At 8-month follow-up, echocardiography demonstrated normalization of left ventricular function and no recurrence or new vascular involvement.

**Discussion:**

The case highlights the importance of considering BS in the differential diagnosis of young patients presenting with acute aortic disease. Aortic involvement in BS, although rare, can lead to severe complications. To the best of our knowledge, this is the first reported case of a pseudoaneurysm of the aortic root as the initial manifestation of BS. Rapid surgical intervention followed by appropriate medical therapy led to a favourable outcome. Recognizing BS as a potential cause of acute presentations of ascending aortic disease may ensure early and effective management.

Learning points

- Ascending aortic pseudoaneurysm is a rare but severe complication of Behçet’s syndrome.
- It may be the first presentation of the disease and the diagnosis can be challenging during the acute phase.

## Introduction

Behçet’s syndrome (BS) is a chronic multisystem inflammatory disorder typically characterized by recurrent oral and genital ulcers and ocular and cutaneous lesions. Vascular involvement, which occurs in over a third of cases, may affect both the arterial and venous systems. Superficial thrombophlebitis and deep vein thrombosis are the most typical vascular manifestations.^1,2^ Arterial disease, although less frequent, can include pseudoaneurysms (PSAs), resulting from prolonged inflammation of the vessel wall. Unlike true aneurysms, pseudoaneurysms are not contained by the vessel wall, increasing susceptibility to rupture with catastrophic consequences.^3^ Arterial pseudoaneurysms can mimic infective endocarditis, posing a significant diagnostic challenge. Herein, we present a rare vascular complication of BS, manifesting as a pseudoaneurysm of the ascending aorta with aortic valve involvement. This case underscores the challenges in diagnosing and managing this life-threatening disease.

## Summary figure

**Figure:**
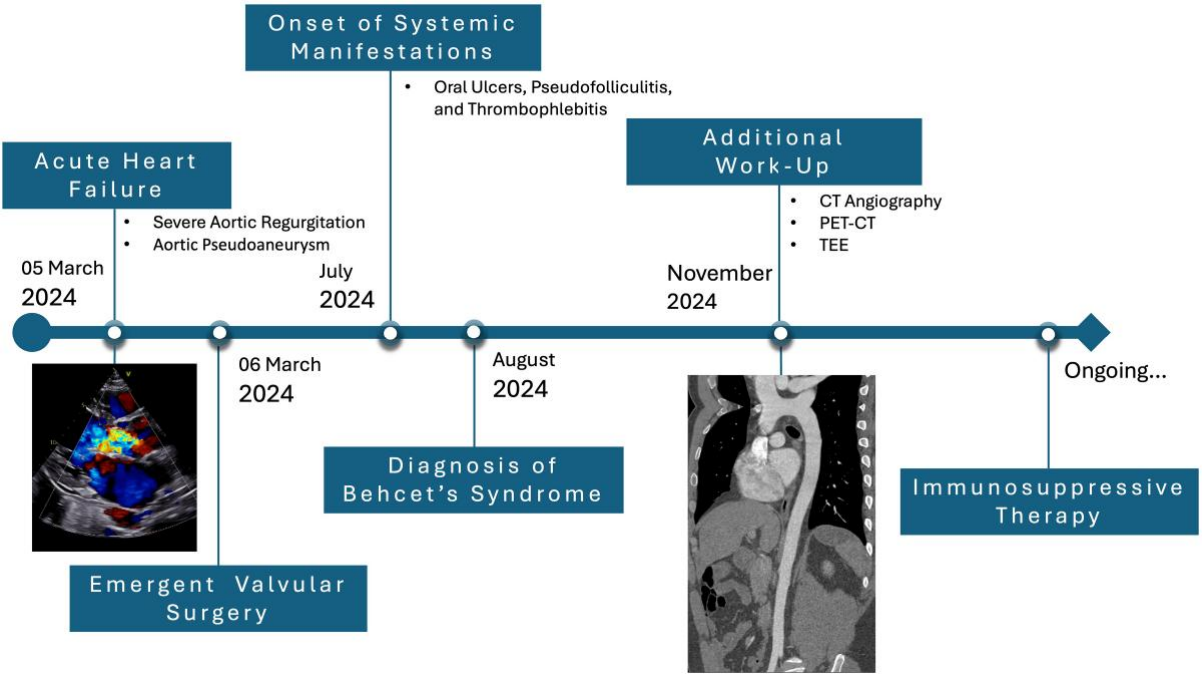


## Case presentation

A 32-year-old patient of sub-Saharan origin without significant known medical or family history was admitted to the emergency department with chest pain, palpitations, and recent onset dyspnoea and orthopnoea. Blood pressure was 150/68 mmHg, heart rate was 99 b.p.m., and oxygen saturation was 97% on room air. Heart rhythm was irregular. A diastolic aortic murmur and bilateral basal hypoventilation were detected.

The electrocardiogram showed left axis deviation and atrial fibrillation. Laboratory findings revealed a haemoglobin 12.9 g/dL (normal: 13–18 g/dL), white blood cell count 5.3 × 10³/µL (normal: 4–10 × 10³/µL), serum creatinine 1.41 mg/dL (normal: 0.6–1.2 mg/dL) with estimated glomerular filtration rate 67 mL/min/1.73m², C-reactive protein 9.3 mg/L (normal < 5 mg/L), and N-terminal pro b-type natriuretic peptide 2820 ng/L (normal: <125 ng/L). Chest X-ray showed cardiomegaly and bilateral pleural effusions. The patient was admitted for acute heart failure and atrial fibrillation, with suspected aortic valve disease.

**Figure 1:**
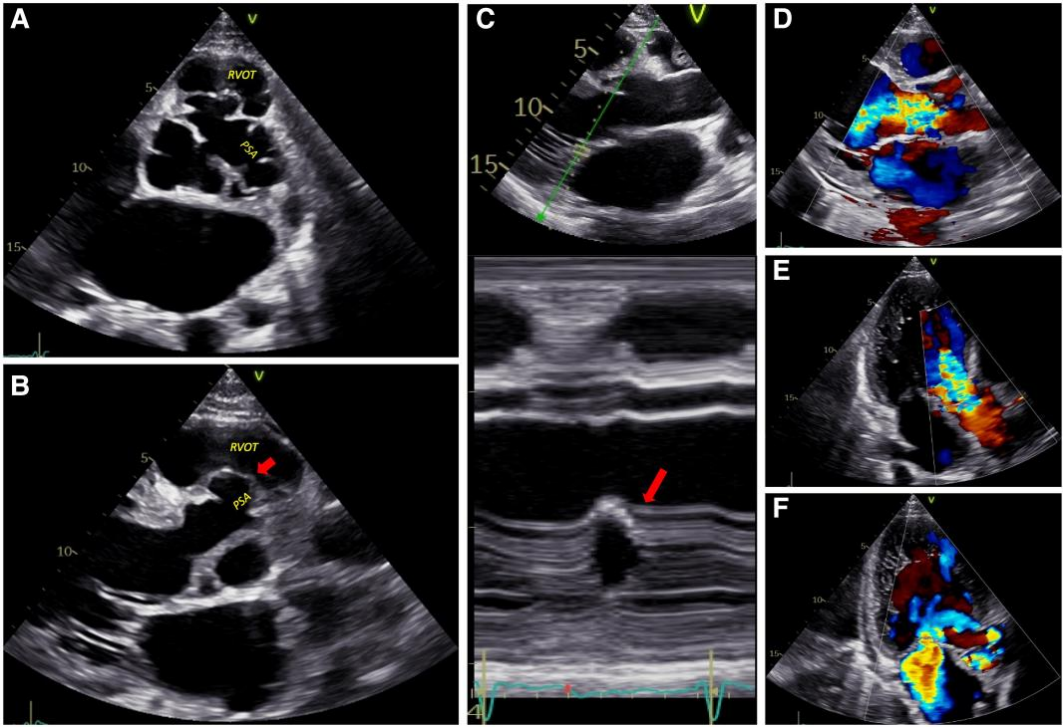
(*A*) Transthoracic parasternal short-axis view showing a multiloculated cavity consistent with a pseudoaneurysm of the ascending aorta. (*B*) Parasternal long-axis view revealing the pseudoaneurysm with a thin wall at the level of the right ventricular outflow tract. (*C*) M-mode echocardiography demonstrating early mitral valve closure, indicative of severe aortic regurgitation. (*D*) Parasternal long-axis view showing severe aortic regurgitation. (*E*) Apical three-chamber view showing severe aortic regurgitation. (*F*) Apical three-chamber view showing moderate mitral regurgitation. PSA, pseudoaneurysm; RVOT, the right ventricular outflow tract.

**Figure 2:**
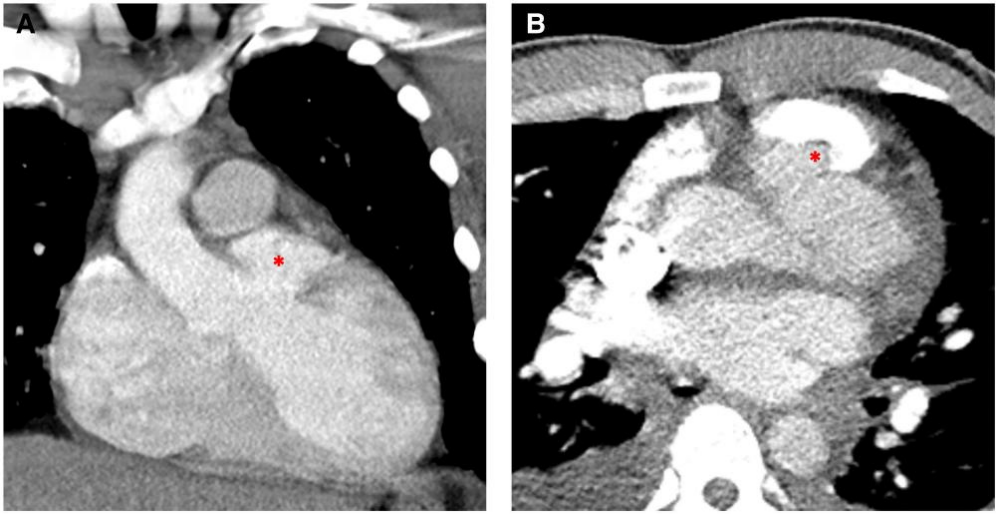
(*A*) Coronal computed tomography reconstruction showing a large pseudoaneurysmal sac (red asterisk) arising from the anterior aspect of the ascending aorta. (*B*) Axial computed tomography confirmed a saccular vascular formation measuring 28 × 33 mm on the left lateral side of the aortic root.

On transthoracic echocardiography, the left ventricle was severely dilated (the end-diastolic and end-systolic diameters were 67 and 48 mm, respectively). Left ventricular ejection fraction was 40%. A multiloculated periaortic cavity was seen at the level of the aortic root, compressing and distorting the aortic valve annulus (*Figure 1A* and *B*, *Video 1*), with severe aortic regurgitation (AR) (*Figure 1D* and *E*, *Video 2*). A thin wall (*Figure 1*, red arrow) separated the pseudoaneurysm from the right ventricular outflow tract (RVOT) suggesting impending rupture. There was no vegetation. The observations of premature mitral valve closure and of a markedly shortened pressure half-time (PHT: 190 ms), were consistent with severely increased end-diastolic left ventricular pressure (LVEDP) (*Figure 1C*). Additionally, moderate to severe functional mitral regurgitation was noted (*Figure 1F*). Cardiac output was preserved, and pulmonary artery systolic pressure (PAP) was estimated at 45–50 mmHg. Contrast-enhanced thoracic computed tomography (CT) confirmed a saccular vascular formation measuring 28 × 33 mm on the left lateral aspect of the aortic root (*Figure 2A*) close to the RVOT (*Figure 2B*), without evidence of aortic dissection or left-to-right shunting. A provisional diagnosis of a periaortic abscess complicated by severe AR and heart failure was made, although no vegetation or other signs of infective endocarditis were present. Antibiotic therapy (ceftriaxone, amoxicillin, and gentamicin) was promptly initiated given the high suspicion of infectious aetiology. The deteriorating patient’s condition and the risk of rupture required emergent surgery.

**Figure 3:**
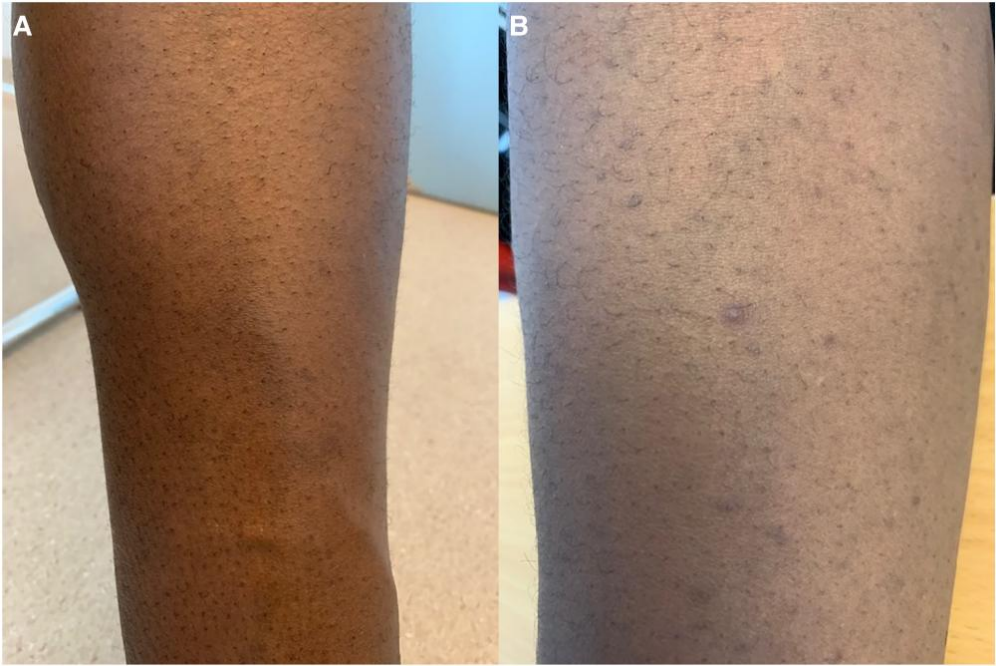
(*A*) Clinical image of the leg showing a palpable superficial venous cord consistent with superficial thrombophlebitis. (*B*) Clinical image of spontaneous pseudofolliculitis on the patient’s skin, consistent with systemic manifestations of Behçet’s syndrome.

Perioperative inspection confirmed a near-ruptured aortic root pseudoaneurysm with compression and distortion of the aortic annulus with thickening of the right and left coronary cusps. A Bentall procedure was performed, including reconstruction of the LVOT using a bovine pericardial patch and implantation of a mechanical prosthetic valve. Postoperative echocardiography (Day 5) showed a well-functioning prosthetic valve without paravalvular leakage and trace mitral regurgitation. Multiple blood cultures taken pre- and post-operatively remained negative. Intraoperative biopsies, including from the valve, revealed non-specific inflammatory changes, and no sign of endocarditis. Serologies for Q fever, brucellosis, syphilis, bartonellosis, and rheumatoid factor were also negative. The patient had an uneventful recovery and was discharged after 3 weeks. During follow-up visits, he reported episodes of spontaneous pseudofolliculitis (*Figure 3A*), swelling of the left leg consistent with superficial venous thrombophlebitis confirmed by Doppler ultrasound (*Figure 3B*), and recurrent oral ulcers. Blood tests showed a mild increase in inflammatory markers and liver enzymes. These findings led to the final diagnosis of BS, complicated by a pseudoaneurysm of the aortic root and acute AR. HLA-B51 testing was negative. Although the patient was a former professional footballer, he did not recall any history of severe thoracic trauma. Neurological and ophthalmological evaluations showed no evidence of central nervous system or ocular involvement. Post-operatively, the patient was started on induction therapy with methylprednisolone 500 mg for 3 days and i.v. infliximab 5 mg/kg. Methylprednisolone 0.75 mg/kg/day was tapered to stop over 6 months while adding azathioprine (2 mg/kg/day) to the maintenance therapy with 6-weekly i.v. infliximab. In addition, heart failure therapy was promptly initiated, including dapagliflozin, bisoprolol, and lisinopril, and continued throughout the 8-month follow-up period. During this follow-up, repeated echocardiography showed no recurrence of AR or abscess. Left ventricular systolic function had normalized (LVEF 55%) with mild end-diastolic dilatation (58 mm). Computed tomography and PET/CT showed no secondary vascular involvement, recurrence, or new pseudoaneurysm.

## Discussion

Behçet’s syndrome is a chronic, recurrent systemic inflammatory disease with complex and pleomorphic clinical manifestations. The prevalence is higher along natives of the historical Silk Road, with the highest prevalence observed in Turkey (up to 420 cases per 100 000 persons).^4,5^

Large and/or medium vessel involvement is seen in ∼40% of the cases, particularly in young male patients. Aortic pseudoaneurysm is a rare but severe vascular complication, resulting from inflammatory destruction of the aortic wall. Behçet’s syndrome-related aneurysms most commonly affect the aorta, pulmonary arteries, and femoral arteries, with aortic involvement carrying the highest risk of rupture.^6,7^ The abdominal aorta, particularly the infra- or suprarenal segments, is the most common site, whereas ascending aorta is among the rarest. It is typically discovered incidentally, either during routine follow-up of the disease or as part of an extension workup, often in asymptomatic patients. The particularity of our case is that decompensated acute AR was the initial manifestation of BS.^8^

The ascending aorta is particularly vulnerable due to high-flow dynamics and valve-related turbulence. Given the unpredictable nature of inflammatory episodes, decisions regarding optimal timing and technique for pseudoaneurysm repair remain a major clinical challenge.^4,9^ In this case, the pseudoaneurysm formed at the left lateral side of the aortic root, with progressive AR and acute heart failure, eventually leading to emergent surgical intervention.

Although BS typically presents with oral and genital mucocutaneous lesions, vascular manifestations (thrombophlebitis, aneurysms, arterial occlusion) can sometimes be the initial presentation, leading to diagnostic delays.^2,4,10^ The absence of validated biomarkers and specific histological features, along with the variability of clinical manifestations contributes to the diagnostic challenge. Although histopathological examination of the aortic wall revealed a predominantly lymphoid inflammatory infiltrate, these findings were considered nonspecific and not diagnostic of BS. Specific histological features of Behçet’s-related vasculitis, including neutrophilic perivasculitis, are extremely rare, and the diagnosis relies primarily on clinical criteria.^5^ HLA-B51 was negative in our patient, but this allele is only present in ∼29.4% of Behçet’s patients of sub-Saharan origin.^11^ Our diagnosis was confirmed by the International Criteria for Behçet’s Disease (ICBD),^3^ which assigns two points for ocular inflammation, oral aphthae, or genital ulcers and one point for cutaneous lesions, vascular manifestations, and central nervous system involvement. A positive pathergy test adds one point. The diagnosis of BS is confirmed when the score is ≥4 points; in our patient, the total score was 4.

Severe AR was central in the clinical presentation. Severe LV dilatation was consistent with previous chronic subclinical AR, but the premature mitral valve closure indicates severely elevated LVEDP and is consistent with acute aggravation of this chronic AR. Hence, although atrial fibrillation may have aggravated the clinical picture, it is likely that the patient would have been symptomatic even in sinus rhythm. Common causes of acute AR include infectious endocarditis (as initially suspected) and Type A aortic dissection. The lack of clinical signs of infection nor of positive blood cultures and of evidence of dissection on thoracic CT allowed ruling out these diagnoses. This case highlights the importance of considering less common diagnosis of acute AR, particularly when associated with aneurysmal disease of the ascending aorta. These include infectious causes (e.g. syphilis), congenital abnormalities (e.g. bicuspid aortic valve), and genetic disorders (e.g. Marfan syndrome and Ehlers–Danlos syndrome), as well as vasculitis and inflammatory diseases (e.g. giant cell arteritis, Takayasu arteritis, and spondyloarthropathies).^12^ In our case, the subsequent discovery of typical systemic manifestations led to the diagnosis of BS. To the best of our knowledge, we herein report the first case of an aortic root pseudoaneurysm with severe acute AR as initial manifestation of BS.

Treatment of aortic pseudoaneurysms in BS requires a multidisciplinary approach, with surgery being central to prevent rupture.^9^ In our patient, the decision to perform emergent surgery was straightforward due to the high risk of rupture and haemodynamic compromise. However, during the inflammatory phase of the disease, the optimal timing of surgery in relation to immunosuppressive treatment is debated. Postoperatively, continued corticosteroids and immunosuppressants are mandatory to control the disease and prevent recurrence. Given the variable manifestations of BS, an individualized approach is essential. As in our case, patients with aortic aneurysms require high-dose glucocorticoids and cyclophosphamide or TNF-α inhibitors should be strongly considered. Infliximab was chosen as an alternative to cyclophosphamide, in line with European Alliance of Associations for Rheumatology recommendations, and supported by Sadoun *et al*., showing higher response rates and better safety.^5,13,14^ To date, imaging exams have revealed no secondary vascular involvement or recurrence. Retrospective data indicate that radiographic progression may appear as late as 2–5 years (22.7% of cases); however, given the higher risk of recurrence, shorter follow-up intervals are warranted in patients with aortic involvement.^15^

## Conclusion

Ascending aortic pseudoaneurysm is a severe but extremely uncommon complication of BS. Because it may be its initial presentation, the diagnosis of BS should be considered when a vascular pseudoaneurysm is discovered, and prompt surgical intervention should be combined with appropriate medical treatment to optimize the outcome.

## Lead author biography


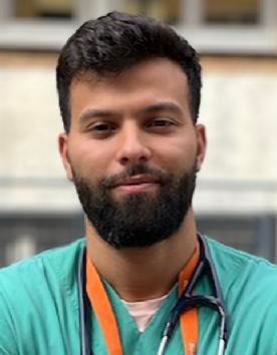


Sohaïb Mansour, MD, is a cardiologist in the Department of Cardiology at CHU Saint-Pierre, Brussels, Belgium. His main interests include cardiovascular imaging and heart failure.

## Data Availability

The data underlying this article will be shared on reasonable request to the corresponding author.
